# Genome-wide copy number variation analysis of hepatitis B infection in a Japanese population

**DOI:** 10.1038/s41439-021-00154-w

**Published:** 2021-06-08

**Authors:** Masataka Kikuchi, Kaori Kobayashi, Nao Nishida, Hiromi Sawai, Masaya Sugiyama, Masashi Mizokami, Katsushi Tokunaga, Akihiro Nakaya

**Affiliations:** 1 Department of Genome Informatics, Graduate School of Medicine, Osaka University, Osaka, Japan; 2 Medical Solutions Division, NEC Corporation, Tokyo, Japan; 3 Genome Medical Science Project, National Center for Global Health and Medicine, Tokyo, Japan; 4 Department of Human Genetics, Graduate School of Medicine, The University of Tokyo, Tokyo, Japan; 5 Present Address: Japanese Red Cross Society, Tokyo, Japan; 6 Present Address: Genome Medical Science Project, National Center for Global Health and Medicine, Tokyo, Japan; 7 Present Address: Laboratory of Genome Data Science, Graduate School of Frontier Sciences, The University of Tokyo, Tokyo, Japan

**Keywords:** Genetics, Medical genomics

## Abstract

Genome-wide association studies have been performed to identify common genetic variants associated with hepatitis B (HB). However, little is known about copy number variations (CNVs) in HB. In this study, we performed a genome-wide CNV analysis between 1830 healthy controls and 1031 patients with HB infection after quality control. Using signal calling by the Axiom Analysis Suite and CNV detection by PennCNV software, we obtained a total of 4494 CNVs across all individuals. The genes with CNVs that were found only in the HB patients were associated with the immune system, such as antigen processing. A gene-level CNV association test revealed statistically significant CNVs in the contactin 6 (CNTN6) gene. Moreover, we also performed gene-level CNV association tests in disease subgroups, including hepatocellular carcinoma patients, liver cirrhosis patients, and HBV carriers, including asymptomatic carriers and patients with HBV-derived chronic hepatitis. Our findings from germline cells suggested that patient-specific CNVs may be inherent genetic risk factors for HB.

## Hepatitis B: Gene copy numbers associated with risk of infection

The risk of contracting the hepatitis B virus may be linked to the number of copies of certain genes in an individual’s genome. A Japanese team led by Masataka Kikuchi, Osaka University, and Akihiro Nakaya, University of Tokyo, looked for repeated segments of the genome, known as copy number variants (CNVs), that differed between people with hepatitis B infections and those without. Studying around 3000 individuals of Japanese descent, the researchers identified several rare CNVs associated with immune function in hepatitis-affected individuals. They also found a common CNV in a gene called *CNTN6* that the hepatitis B virus often uses to integrate itself into the genome of liver cells, a process that can lead to cancer. The findings underscore the importance of CNVs as inherited risk factors for hepatitis B and other viral infections.

## Introduction

Hepatitis B (HB) is an infectious disease caused by the HB virus (HBV). The World Health Organization (WHO) estimated that 257 million people have chronic HBV infection. HBV infection leads to liver inflammation and can subsequently cause hepatocellular carcinoma (HCC) and liver cirrhosis (LC); in particular, HCC mainly occurs in sub-Saharan Africa and Eastern Asia, including Japan^1^.

To identify the inheritance of a predisposition for HB, genome-wide association studies (GWASs) have been performed using germline tissues such as blood cells in Chinese, Korean, and Japanese populations^2–8^. These GWASs have reported that some susceptibility loci are located in human leukocyte antigen (HLA) regions, including HLA-DPB1, HLA-DPA1, and HLA-DQB2. In addition, a GWAS of an Indonesian population revealed that single nucleotide polymorphisms (SNPs) in the HLA regions contributed to the reactivity to the HB vaccine^9^. HLA presents antigens, including those produced during viral infections, to T cells and regulates the immune system. This result suggests that immune system variations in individuals are associated with the pathogenesis of HB. However, little is known about the contribution of structural variants such as copy number variations (CNVs) to HB.

Genomic DNA is usually present in two copies in a human cell, whereas its copy number changes by genome duplication and deletion events. These DNA segments differing in copy number in a population are called CNVs. CNVs can affect gene expression and are associated with the development of disease.

In somatic cells, the integration of HBV into the genome of human hepatocytes increases chromosomal instability and has the potential to alter the copy number of human DNA^10^. Indeed, the number of somatic CNVs in liver tissues of patients with HCC was positively correlated with the number of HBV integrations into the human genome^10^. Liver tissues from patients with LC have more CNVs than those from healthy participants^11^. These studies suggested that the accumulation of somatic CNVs reflects the severity of the pathology and the degree of HBV integration.

In the germline, Clifford et al. examined CNVs in 386 patients with HCC in Korean and Chinese populations and identified CNVs in the ALDH7A1, C4orf29, C5orf48, KNG1, LARP2, LMNB1, PHAX, PUS7, SRPK2, and TMPO genes^12^. Some of these CNVs were related to the immune response and tumorigenesis. However, there have been no large-scale studies of germline CNVs in the Japanese population.

In this study, we performed genome-wide CNV analysis of more than 3000 Japanese individuals to detect germline CNVs. Peripheral blood samples were assayed for CNVs using Axiom Genome-Wide ASI 1 Array Plates optimized to detect not only common variants but also rare variants in the East Asian population. Using signal calling by the Axiom Analysis Suite and CNV detection by PennCNV software, we obtained a total of 4494 CNVs across all individuals. We also calculated various statistics between the case and control individuals and between different disease types, including HCC and LC. The CNV data and statistics from this study will serve as a resource for clarifying the genetic predisposition to HB.

## Materials and methods

### Subjects

Subjects including healthy controls (*n* = 2097; 31.0 ± 12.2 years old, based on 1514 subjects with age data) and HBV patients (*n* = 1061; 57.8 ± 12.8 years old, based on 601 subjects with age data) were recruited at 28 multicenter hospitals (liver units with hepatologists) and universities throughout Japan. These subjects were included in previous studies^13,14^. HBV status was determined based on the serological results for HB surface antigen (HBsAg) and HB core antibody (anti-HBc) using a fully automated chemiluminescent enzyme immunoassay system (Abbott ARCHITECT; Abbott Japan, Tokyo, Japan, or LUMIPULSE f or G1200; Fujirebio, Inc., Tokyo, Japan). Unrelated and anonymized healthy control samples were collected from Japanese volunteers with/without HBV vaccination. HBV patients included HBV-positive HCC patients (*n* = 488), HBV-positive LC patients (*n* = 46), and HBV carriers, including asymptomatic carriers (ASCs) and individuals with HBV-derived chronic hepatitis (CH) (ASC + CH, *n* = 527).

### Genotyping and quality control

GWAS genotyping was performed using the Affymetrix Axiom Genome-Wide ASI 1 Array (Thermo Fisher Scientific, Inc., Waltham, MA, USA), which contains 600,307 markers corresponding to common and rare alleles in a consensus East Asian (including Japanese) genome. First, we excluded poor-quality samples and genotyped the markers of the samples that passed quality control (QC) using the Axiom™ Analysis Suite. The thresholds for QC were the default thresholds in the Axiom™ Analysis Suite: dish QC threshold ≥0.82; QC call rate threshold ≥97%; plate QC percent samples passed ≥95%; and plate QC average call rate ≥98.5%. All individuals who passed QC were examined for potential genetic relatedness by calculating identity-by-descent estimates for all possible pairs of individuals in PLINK. We removed one individual with a low QC call rate within each pair according to pi-hat (>0.4). Finally, we obtained 1831 healthy controls and 1042 HBV patients. We identified CNVs using 345,987 markers that satisfied the following thresholds: minor allele frequency >0.10; missing call rate <1%; and Hardy–Weinberg equilibrium *p*-value >1.0 × 10^−6^.

### CNV calls

CNVs from the samples were called using PennCNV^15^. PennCNV uses the log R ratio (LRR) value and the B allele frequency (BAF) for each SNP to infer the copy number states of each SNP. LRR indicates a normalized measure of the total signal intensity of the B and A alleles and directly reflects an increase or decrease in the copy number. The BAF shows a normalized measure of the relative signal intensity ratio of the B and A alleles and helps differentiate copy number states (e.g., differentiate copy-neutral loss of heterozygosity regions and normal state regions). PennCNV calculates the probability of observing a particular copy number state by the hidden Markov model (HMM), given the LRR and BAF for each SNP. A population frequency of the B allele (PFB) file and a GC model file were generated from 1831 healthy controls using compile_pfb.pl and cal_gc_snp.pl in PennCNV. An HMM file was provided by Thermo Fisher Scientific, Inc. Only samples with a standard deviation of the log R ratio with a normalized intensity <0.35, B allele frequency drifting value <0.01, and wave factor value between −0.05 and 0.05 were analyzed. Adjacent CNVs separated by a gap of <20% of the combined length of the two CNVs were merged until no more gaps of <20% existed, and CNVs based on fewer than 5 markers were excluded. In this process, we examined four cutoffs in terms of the number of markers included in a CNV, which were >5, >10, >15, and >20 markers. Several genomic regions are known to harbor spurious CNV calls. We excluded centromeric regions, telomeric regions, segmental duplication regions, immunoglobulin regions, and repeat-masked regions. These regions were provided by PennCNV (http://penncnv.openbioinformatics.org/en/latest/misc/faq/). The immunoglobulin regions included four regions (chr2:88937989–89411302, chr14:21159897–22090937, chr14:105065301–106352275, and chr22:20715572–21595082). These regions were transformed from the reference genome hg18 to hg19 using the UCSC LiftOver tool (http://genome.ucsc.edu/cgi-bin/hgLiftOver). We also excluded T-cell receptor (TCR) and immunoglobulin heavy (IGH) chain genomic regions from our analyses because these regions undergo V-(D)-J recombination in lymphocytes and can yield somatic CNVs rather than germline CNVs^16^. These regions included TCR alpha and delta on chromosome 14 (chr14:22090057–23021075 and chr14:22891537–22935569, respectively), beta and gamma on chromosome 7 (chr7:141998851–142510972 and chr7:38279625–38407656, respectively), and IGH regions on chromosomes 14 and 16 (chr14:106032614–107288051 and chr16:33740716–33741266). Individuals of unknown sex were eliminated. After CNV calls, we finally identified 1830 healthy controls and 1031 HBV patients. Only autosomes were analyzed. PennCNV classifies CNV events according to six state definitions: state 1 = deletion of two copies (copy number: 0), state 2 = deletion of one copy (copy number: 1), state 3 = two-copy state (copy number: 2), state 4 = two-copy state with loss of heterozygosity (copy number: 2), state 5 = duplication of one copy (copy number: 3), and state 6 = duplication of two copies (copy number: 4). A copy number of two was considered normal. CNVs with a copy number >2 were defined as duplications, while those with a copy number <2 were considered deletions. The distribution of CNVs was drawn by the R package “RIdeogram”^17^.

### Statistical tests for global burden and gene-level CNV association analyses

Statistics were calculated by permutation tests (50,000 random permutations for the global burden test and 10,000 random permutations for the gene-level CNV association test). The permutation tests were performed using the following procedures: (i) the case-control label of each subject was shuffled, and (ii) the empirical *p*-value of the *j*th marker was calculated as (*R* + 1)/(*N* + 1), where *N* is the number of permutations and *R* is the number of times the permuted test statistics (i.e., *S*_*j,*1_ ~ *S*_*j,N*_) were greater than the observed statistics of the *j*th marker *S*_*j,*obs_. *P*-values were adjusted by the max(T) procedure to regulate the familywise error rate^18^. The max(T) procedure was performed as follows: (i) the largest statistic of the *j*th marker *S*_*j,*max_ was selected from the statistics of the permuted data sets, and (ii) an adjusted *p*-value was calculated as (*R* + 1)/(*M* + 1), where *M* is the number of markers and *R* is the number of times the permuted test statistics (i.e., *S*_1*,*max_ ~ *S*_*M,*max_) were greater than *S*_*j,*obs_. For gene-level CNV association tests, the gene region was used for UCSC-known gene annotation (knownGene.txt.gz and kgXref.txt.gz). Each test was performed by PLINK software (PLINK command for the global burden test: --cnv-indiv-perm --mperm 50000; PLINK command for the gene-level CNV association test: --cnv-intersect genelist --cnv-test-region --mperm 10000)^19^. In this study, we defined a nonadjusted *p*-value <0.05 as nominally significant.

### Gene functional enrichment analysis

We used the Molecular Signatures Database (MSigDB) in Gene Set Enrichment Analysis (GSEA) and Metascape software to examine the functions of genes^20–22^. We examined overlaps with Gene Ontology (GO) biological processes in the GSEA-MSigDB analysis. HBV patient-unique genes were defined as genes with one or more CNV in HBV patients and no CNVs in the healthy controls. In contrast, the healthy control-unique genes were defined as the genes found to have more than one CNV in the healthy controls and none found in the HBV patients.

### Genomic regulatory regions for 127 tissues or cell types

Genomic regulatory regions for 127 tissues or cell types were obtained from the Roadmap Epigenomics website (http://egg2.wustl.edu/roadmap/web_portal/). The chromatin state model segments the human genome into 25 states based on 12 chromatin marks (H3K4me1, H3K4me2, H3K4me3, H3K9ac, H3K27ac, H4K20me1, H3K79me2, H3K36me3, H3K9me3, H3K27me3, H2A.Z, and DNase I-hypersensitive sites) using ChromHMM and ChromImpute^23,24^. We grouped these states into the following 12 subgroups: active TSS (TssA), promoter (PromU, PromD1, and PromD2), transcribed (Tx5, Tx, Tx3, and TxWk), transcribed and regulatory (TxReg, TxEnh5, TxEnh3, and TxEnhW), active enhancer (EnhA1, EnhA2, and EnhAF), weak enhancer (EnhW1, EnhW2, and EnhAc), DNase (DNase), ZNF genes and repeats (ZNF/Rpts), heterochromatin (Het), poised promoter (PromP), bivalent promoter (PromBiv), and repressed polycomb (ReprPC). The DNA segments in these 12 subgroups from each tissue or cell type are described in bed files. We compared the number of genomic regulatory regions included in the CNVs between the healthy controls and the HBV patients with the following PLINK command: --cnv-indiv-perm --mperm 50000 --cnv-count bedfile.

## Results

### Global CNV burden analysis

**Fig. 1:**
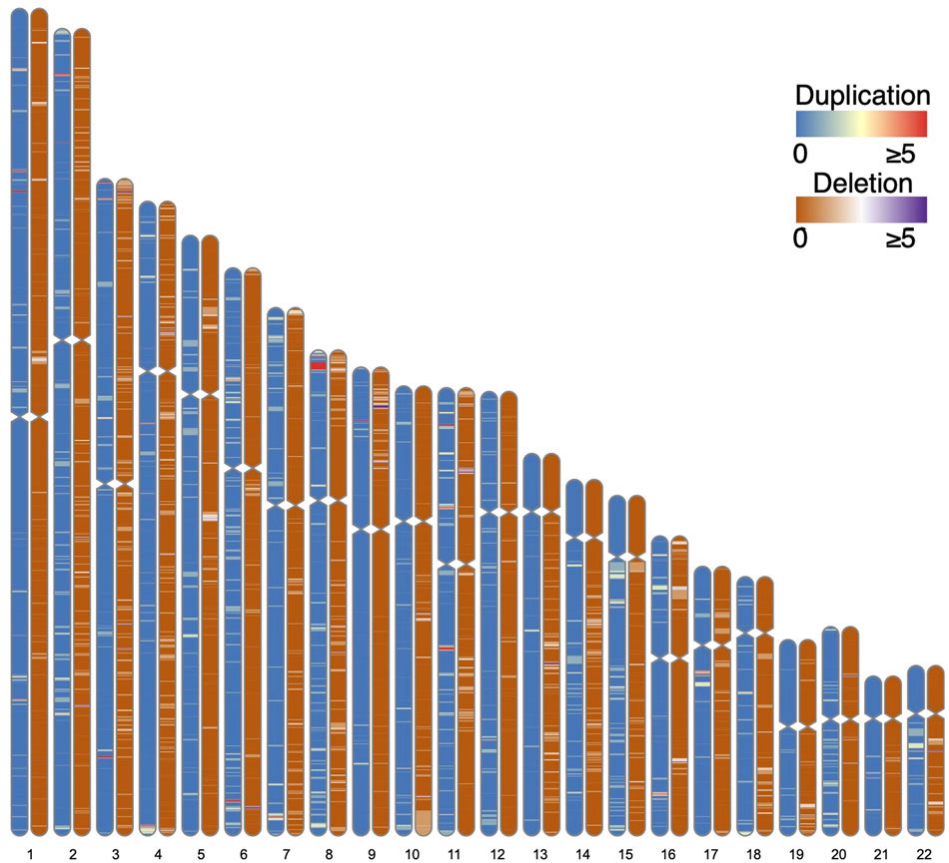
The distribution of CNVs. Heatmaps indicate the number of subjects having CNVs with >5 markers.

We performed global CNV burden analysis using four different CNV cutoffs to examine the characteristics of CNVs between 1830 healthy controls and 1031 HBV patients. We detected 2098 CNVs with a cutoff of >20 markers and 4470 CNVs with a cutoff of >5 markers in the healthy controls and HBV patients (Fig. 1, Table 1). The proportion of samples with one or more CNV in HBV patients (74.6%) was higher than that in the healthy controls (68.5%) for a cutoff of >5 markers (adjusted *p*-value = 1.84 × 10^−3^), although there was no significant difference for the other cutoffs (Table 1). The number of CNVs with at least one gene in the HBV patients (0.552) was higher than that in the healthy controls (0.498) for a cutoff of >5 markers (adjusted *p*-value = 1.27 × 10^−2^). This difference was not significant for the other cutoffs, suggesting that this tendency depended on the proportion of samples with one or more CNV.

**Table 1:** Global CNV burden analysis.

		>5 markers			>10 markers			>15 markers			>20 markers		
		CASE	CTRL	Adjusted *P*-value	CASE	CTRL	Adjusted *P*-value	CASE	CTRL	Adjusted *P*-value	CASE	CTRL	Adjusted *P*-value
Total number of samples with CNV(s)		769	1253	−	661	1111	−	550	930	−	471	788	−
Total number of deletions	Copy number 0	4	6	−	3	2	−	3	1	−	3	1	−
	Copy number 1	1049	1896	−	745	1528	−	480	1072	−	341	764	−
Total number of duplications	Copy number 3	405	938	−	339	843	−	307	747	−	286	684	−
	Copy number 4	49	123	−	12	28	−	7	18	−	5	14	−
Proportion of sample with one or more CNVs	74.6%	68.5%	**1.84** × **10**^**−3**^	64.1%	60.7%	0.160	53.4%	50.8%	0.416	45.7%	43.1%	0.380	
Average size of CNV (kb)		220.1	207.9	0.856	286.6	255.3	0.268	360.3	315.7	0.180	420.6	370.0	0.220
Number of CNVs with at least one gene		0.552	0.498	**1.27** × **10**^**−2**^	0.461	0.424	0.128	0.395	0.373	0.548	0.359	0.325	0.144
Number of genes per 100 kb CNV		1.151	1.006	0.504	0.775	0.786	1.000	0.689	0.774	1.000	0.692	0.775	1.000

Bonferroni-adjusted *P*-value <0.05 is indicated in bold. *P*-values were calculated by 50,000 random permutations in plink software.

We identified 1153 genes with CNVs for a cutoff of >5 markers in the HBV patients. Among these genes, 751 were unique to the HBV patients. We next performed gene functional enrichment analysis using GSEA software to identify the functions of these HBV patient-unique genes with CNVs. As a result, 22q11.2 deletion syndrome (false-discovery rate (FDR)-adjusted *p*-value = 6.82 × 10^−27^), steroid hormone biosynthesis (FDR-adjusted *p*-value = 4.54 × 10^−4^), and antigen processing and presentation (FDR-adjusted *p*-value = 1.62 × 10^−3^) were significantly associated with the HBV patient-unique genes (Table S1). Metascape software also revealed these associations (FDR-adjusted *p*-value < 0.05) (Table S2). Interestingly, the genes with CNVs found in the healthy controls were not associated with these terms (Tables S3 and S4).

### Genomic regulatory regions covered by CNVs

**Fig. 2:**
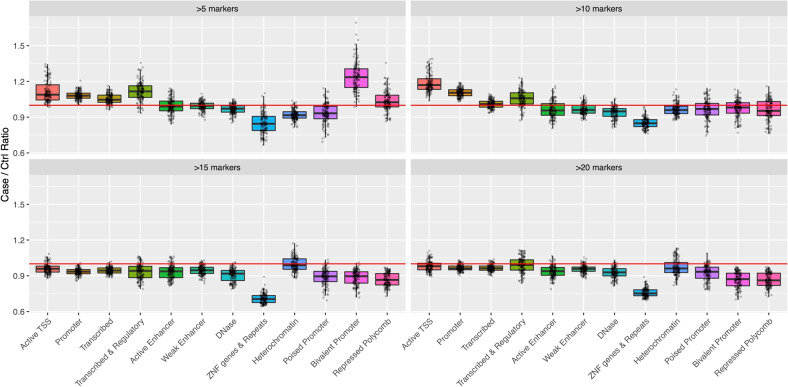
The case-control ratio of genomic regulatory regions per 100 kb CNV in the 12 subgroups. Each plot indicates the case-control ratio in a tissue or cell line. There were 127 plots corresponding to tissues or cell lines in each subgroup.

We next focused on the genomic regulatory regions activated in different tissues and investigated those included in the CNVs in the HBV patients. The number of genomic regulatory regions included in the CNVs was compared between the healthy controls and the HBV patients using a chromatin state model for 127 tissues or cell types from Roadmap Epigenomics. We divided the genomic regulatory regions into 12 subgroups (see the “Materials and methods” section). Figure 2 shows the number of genomic regulatory regions per 100 kb CNV in each subgroup as the ratio between the HBV patients and the healthy controls. With a cutoff of >5 markers, the CNVs in the HBV patients were primarily dominated by six subgroups, including promoters and transcribed regions, compared with those in the healthy controls (active TSS, promoter, transcribed, transcribed & regulatory, bivalent promoter, and repressed polycomb in Fig. 2). Some trends were also observed with a cutoff of >10 markers but not with cutoffs of >15 and >20 markers, indicating that CNVs with a relatively small size covered the promoter and transcribed regions. As expected, with a cutoff of >5 markers, CNVs in the HBV patients nominally significantly covered the TSSs and promoters activated in some cell types in our sample, peripheral blood, compared with the healthy controls, although the FDR-adjusted *p*-values did not reach significance (nonadjusted *p*-value < 0.05) (Table S5).

### Gene-level CNV association test

**Fig. 3:**
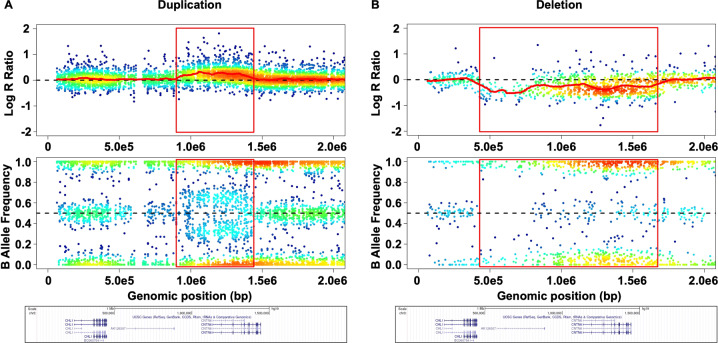
Point density plots of the log R ratio and B allele frequency for duplications (**A**) and deletions (**B**) of the CNTN6 gene. Each duplication shows low LRR (upper panel), and BAF values clustered around 0 (AAA), 0.33 (AAB), 0.66 (ABB), and 1 (BBB) (bottom panel). We did not find 4-copy duplications in this region. Each deletion shows low LRR (upper panel), and BAF values clustered around 0 or 1 (bottom panel).

To search for CNVs associated with HBV, we performed a gene-level CNV association test between the healthy controls and the HBV patients. We found a significant association between HB and the CNVs in the contactin 6 (CNTN6) gene for all cutoffs (adjusted *p*-value < 0.05) (Fig. 3 and S1, Table 2). We also found 13 genes that were nominally associated with HBV with at least one cutoff level (nonadjusted *p*-value <0.05). All CNV regions were found in the HBV patients rather than in the healthy controls. Therefore, the odds ratios (ORs) were positive only when calculatable.

**Table 2:** Gene-based CNV association test of the HBV patients.

		>5 markers					>10 markers					>15 markers					>20 markers				
Gene	Chr:Start–End	Case (Del/Dup)	Ctrl (Del/Dup)	*P*-value	Adjusted *P*-value	OR (95%CI)	Case (Del/Dup)	Ctrl (Del/Dup)	*P*-value	Adjusted *P*-value	OR (95%CI)	Case (Del/Dup)	Ctrl (Del/Dup)	*P*-value	Adjusted *P*-value	OR (95%CI)	Case (Del/Dup)	Ctrl (Del/Dup)	*P*-value	Adjusted *P*-value	OR (95%CI)
SYT11	1:155,829,259–155,854,990	4/0	0/0	0.018	0.666	NA	0/0	0/0	1.000	1.000	NA	0/0	0/0	1.000	1.000	NA	0/0	0/0	1.000	1.000	NA
RIT1	1:155,867,598–155,881,193	4/0	0/0	0.018	0.666	NA	0/0	0/0	1.000	1.000	NA	0/0	0/0	1.000	1.000	NA	0/0	0/0	1.000	1.000	NA
SCARNA4	1:155,895,748–155,895,877	4/0	0/0	0.018	0.666	NA	0/0	0/0	1.000	1.000	NA	0/0	0/0	1.000	1.000	NA	0/0	0/0	1.000	1.000	NA
RXFP4	1:155,911,479–155,912,625	4/0	0/0	0.018	0.666	NA	0/0	0/0	1.000	1.000	NA	0/0	0/0	1.000	1.000	NA	0/0	0/0	1.000	1.000	NA
ARHGEF2	1:155,916,629–155,959,864	4/0	0/0	0.018	0.666	NA	0/0	0/0	1.000	1.000	NA	0/0	0/0	1.000	1.000	NA	0/0	0/0	1.000	1.000	NA
SSR2	1:155,978,838–155,990,758	3/0	0/0	0.047	0.995	NA	0/0	0/0	1.000	1.000	NA	0/0	0/0	1.000	1.000	NA	0/0	0/0	1.000	1.000	NA
ZAP70	2:98,330,030–98,356,323	3/0	0/0	0.042	0.995	NA	3/0	0/0	0.046	0.986	NA	0/0	0/0	1.000	1.000	NA	0/0	0/0	1.000	1.000	NA
TMEM131	2:98,372,800–98,612,354	3/0	0/0	0.042	0.995	NA	3/0	0/0	0.046	0.986	NA	0/0	0/0	1.000	1.000	NA	0/0	0/0	1.000	1.000	NA
CNTN6	3:1,134,619–1,445,278	2/6	0/1	0.001	**0.047**	14.30 (1.79–114.52)	2/6	0/1	0.002	**0.035**	14.30 (1.79–114.52)	2/6	0/1	0.002	**0.031**	14.30 (1.79–114.52)	2/6	0/1	0.002	**0.027**	14.30 (1.79–114.52)
GRIK2	6:101,841,381–102,517,958	1/2	0/0	0.049	0.995	NA	1/2	0/0	0.046	0.986	NA	0/1	0/0	0.363	1.000	NA	0/1	0/0	0.367	1.000	NA
DPP6	7:153,584,418–154,685,995	0/46	2/54	0.033	1.000	1.48 (0.99–2.20)	0/46	2/54	0.036	0.997	1.48 (0.99–2.20)	0/46	0/54	0.024	0.975	1.54 (1.03–2.29)	0/46	0/54	0.025	0.936	1.54 (1.03–2.29)
MAL2	8:120,220,609–120,257,914	0/6	0/0	0.002	0.070	NA	0/2	0/0	0.124	1.000	NA	0/1	0/0	0.366	1.000	NA	0/1	0/0	0.359	1.000	NA
CAMKK2	12:121,675,494–121,736,111	4/0	0/0	0.015	0.666	NA	2/0	0/0	0.128	1.000	NA	0/0	0/0	1.000	1.000	NA	0/0	0/0	1.000	1.000	NA
SNX29	16:12,070,601–12,668,146	4/1	1/0	0.026	0.788	8.91 (1.04–76.40)	4/1	1/0	0.028	0.685	8.91 (1.04–76.40)	2/1	1/0	0.137	1.000	5.34 (0.55–51.38)	0/1	0/0	0.371	1.000	NA

Adjusted *P*-value <0.05 is indicated in bold. *P*-values were calculated by 10,000 random permutations in plink software and were adjusted by the max(T) procedure.

We next analyzed the CNVs in each disease subgroup. The HBV patients were divided into three disease subgroups: HCC, LC, and ASC + CH patients. CNVs with nominally significant differences for at least one cutoff level were located in 27, 42, and 11 genes in HCC, LC, and ASC + CH patients, respectively (Tables S6–S8). The CNVs in CNTN6, which were significant in the above CNV association test when using all HBV patients, were found in four patients each from the HCC and ASC + CH patient subgroups (Tables S6 and S8). After using 146 age-matched controls aged more than 50 years old (58.1 ± 6.45 years old in the healthy controls and 57.8 ± 12.8 years old in the HBV patients), these CNVs were found only in the patients and not in the controls, but the difference was not statistically significant (Table 3).

**Table 3:** Association test of CNTN6 using 146 age-matched controls.

	>5 markers					>10 markers					>15 markers					>20 markers				
Patient	Case (Del/Dup)	Ctrl (Del/Dup)	*P*-value	Adjusted *P*-value	OR (95%CI)	Case (Del/Dup)	Ctrl (Del/Dup)	*P*-value	Adjusted *P*-value	OR (95%CI)	Case (Del/Dup)	Ctrl (Del/Dup)	*P*-value	Adjusted *P*-value	OR (95%CI)	Case (Del/Dup)	Ctrl (Del/Dup)	*P*-value	Adjusted *P*-value	OR (95%CI)
All HBV	2/6	0/0	0.389	0.967	NA	2/6	0/0	0.389	0.917	NA	2/6	0/0	0.383	0.872	NA	2/6	0/0	0.388	0.856	NA
HCC	0/4	0/0	0.380	0.995	NA	0/4	0/0	0.372	0.984	NA	0/4	0/0	0.376	0.896	NA	0/4	0/0	0.378	0.934	NA
ASC + CH	2/2	0/0	0.409	0.994	NA	2/2	0/0	0.412	0.976	NA	2/2	0/0	0.408	0.957	NA	2/2	0/0	0.413	0.893	NA

*P*-values were calculated by 10,000 random permutations in plink software and were adjusted by the max(T) procedure.

We finally compared the CNVs between the three disease subgroups: HCC vs. LC patients, HCC vs. ASC + CH patients, and LC vs. ASC + CH patients. We did not find significant associations in the comparisons of HCC vs. ASC + CH and LC vs. ASC + CH patients. However, in the comparison of HCC vs. LC patients, the CNVs in collectin 10 (COLEC10) were nominally significant with a cutoff of >5 markers (Table S9).

## Discussion

In this study, we examined germline CNVs in Japanese HBV patients. We found that the CNVs in CNTN6 were associated with the HBV patients. To the best of our knowledge, our study examines germline CNVs in HBV patients using the most large-scale genomic dataset in the Japanese population.

We showed that the CNVs in the HBV patients tended to accumulate in genes that play a role in immune system function, such as antigen processing, although we excluded TCR and IGH chain genomic regions, which are regions that dynamically change DNA structure in response to several pathogens (see “Materials and methods”) (Tables S1 and S2). The term antigen processing included the following genes: HLA-A, HLA-DRA, PSMB8, TAP1, and TAP2. The PSMB8, TAP1, and TAP2 genes were located near each other on chromosome 6 and were within the same CNV, which may be significant. Our findings suggest that the immune system is potentially impaired in some HBV patients. Figure 2 shows that the CNVs in the HBV patients were included in promoters and transcribed regions, suggesting that they directly affect the expression levels of genes (e.g., those in the immune system).

In the gene-level CNV association test of the HBV patients, the CNV region covering CNTN6 was statistically significant across all cutoffs (Table 2). CNTN6 belongs to the immunoglobulin superfamily and is implicated in neural developmental events, including neural cell adhesion and migration, neurite outgrowth and fasciculation, axon guidance, and myelination^25–27^. A study showed that HBV DNA integrated into CNTN6 in HBV-related HCC tissues^28^.

In the gene-level CNV association test between HCC and LC patients, we detected COLEC10 with a cutoff of >5 markers (Table S9). COLEC10 is also called collectin liver 1 (CL-L1) and is mainly expressed in the liver. Members of the collectin family, which belongs to the lectin complement pathway, recognize ligands on pathogens, including viruses and bacteria, and drive phagocytosis. We detected duplication of COLEC10 in two LC patients, suggesting the upregulation of COLEC10 (Table S9). A study reported that the protein levels of COLEC10 in the blood of patients with acute liver failure and alcoholic cirrhosis were higher than those in the blood of healthy controls^29^.

Our study has several limitations. First, we detected statistically significant CNVs in CNTN6, but their associations were not significant after using the age-matched controls. Second, most of the CNVs that we found were observed in only a few patients, leading to small effect sizes. Indeed, the 95% confidence interval of most CNVs had a broad range (Table 2). Third, the sample size of the LC patients was extremely small compared with those of the HCC and ASC + CH patients. To overcome these limitations, we must collaborate with international institutes and validate and replicate our findings using more age-matched samples.

## Supplementary information

TableS

FigureS1
