# Supplementary figures and images for: Genome-wide copy number variation analysis of hepatitis B infection in a Japanese population
Source: Hum Genome Var. 2021 Jun 8;8:22. doi: 10.1038/s41439-021-00154-w (PMC8187437; doi:10.1038/s41439-021-00154-w)

**FigureS1**

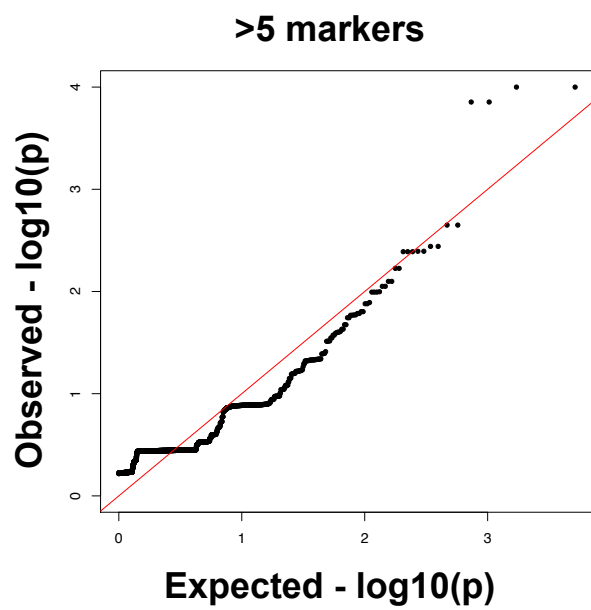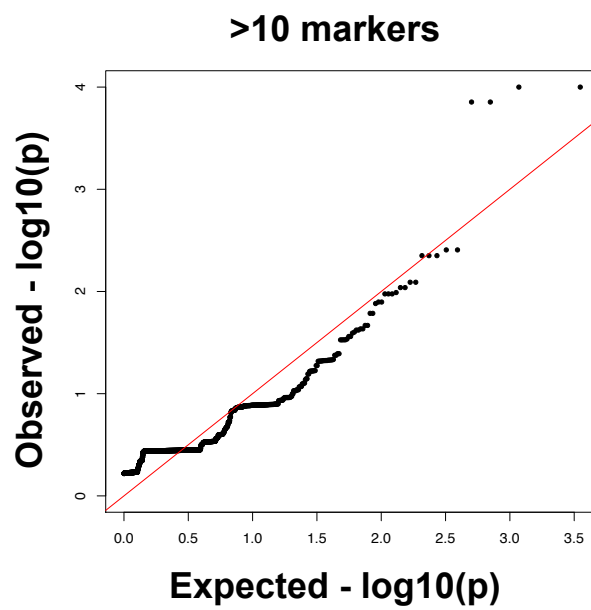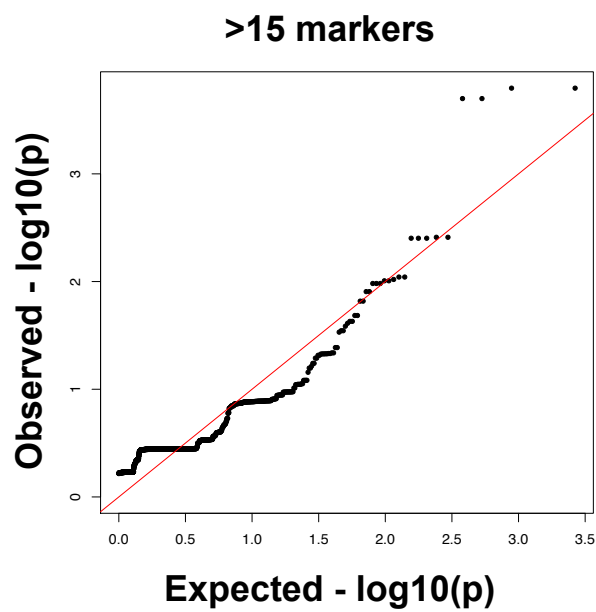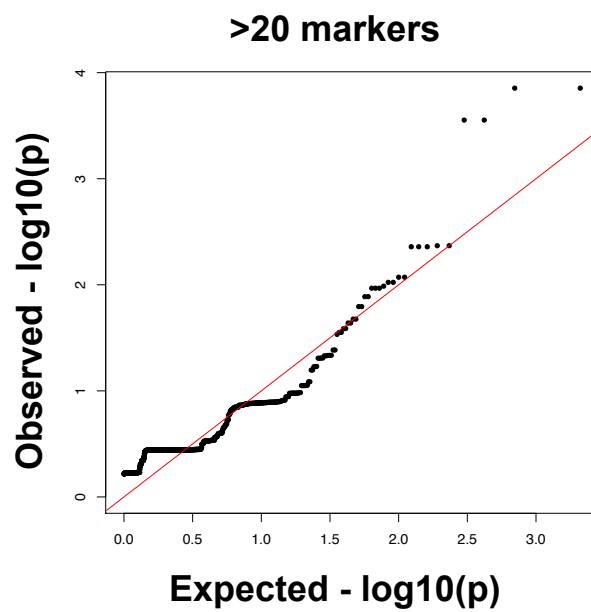

Supplement: Supplementary file 2 — FigureS1 [file 41439_2021_154_MOESM2_ESM.pdf]
